# Matrix Gla protein (MGP), GATA3, and TRPS1: a novel diagnostic panel to determine breast origin

**DOI:** 10.1186/s13058-022-01569-1

**Published:** 2022-10-25

**Authors:** Tian Du, Lu Pan, Chengyou Zheng, Keming Chen, Yuanzhong Yang, Jiewei Chen, Xue Chao, Mei Li, Jiabin Lu, Rongzhen Luo, Jinhui Zhang, Yu Wu, Jiehua He, Dongping Jiang, Peng Sun

**Affiliations:** 1 State Key Laboratory of Oncology in South China, Collaborative Innovation Center for Cancer Medicine, Guangzhou, 510060 People’s Republic of China; 2 Department of Pathology, Sun Yat-Sen University Cancer Center, Guangzhou, 510060 People’s Republic of China; 3 Department of Breast Surgery, Sun Yat-Sen University Cancer Center, Guangzhou, 510060 People’s Republic of China; 4 Department of Medical Imaging, Sun Yat-Sen University Cancer Center, Guangzhou, 510060 People’s Republic of China

**Keywords:** Breast carcinoma, Immunohistochemical, MGP, TRPS1, GATA3

## Abstract

**Background:**

Metastatic breast carcinoma is commonly considered during differential diagnosis when metastatic disease is detected in females. In addition to the tumor morphology and documented clinical history, sensitive and specific immunohistochemical (IHC) markers such as GCDFP-15, mammaglobin, and GATA3 are helpful for determining breast origin. However, these markers are reported to show lower sensitivity in certain subtypes, such as triple-negative breast cancer (TNBC).

**Materials and methods:**

Using bioinformatics analyses, we identified a potential diagnostic panel to determine breast origin: matrix Gla protein (MGP), transcriptional repressor GATA binding 1 (TRPS1), and GATA-binding protein 3 (GATA3). We compared MGP, TRPS1, and GATA3 expression in different subtypes of breast carcinoma of (*n* = 1201) using IHC. As a newly identified marker, MGP expression was also evaluated in solid tumors (*n* = 2384) and normal tissues (*n* = 1351) from different organs.

**Results:**

MGP and TRPS1 had comparable positive expression in HER2-positive (91.2% vs. 92.0%, *p* = 0.79) and TNBC subtypes (87.3% vs. 91.2%, *p* = 0.18). GATA3 expression was lower than MGP (*p* < 0.001) or TRPS1 (*p* < 0.001), especially in HER2-positive (77.0%, *p* < 0.001) and TNBC (43.3%, *p* < 0.001) subtypes. TRPS1 had the highest positivity rate (97.9%) in metaplastic TNBCs, followed by MGP (88.6%), while only 47.1% of metaplastic TNBCs were positive for GATA3. When using MGP, GATA3, and TRPS1 as a novel IHC panel, 93.0% of breast carcinomas were positive for at least two markers, and only 9 cases were negative for all three markers. MGP was detected in 36 cases (3.0%) that were negative for both GATA3 and TRPS1. MGP showed mild-to-moderate positive expression in normal hepatocytes, renal tubules, as well as 31.1% (99/318) of hepatocellular carcinomas. Rare cases (0.6–5%) had focal MGP expression in renal, ovarian, lung, urothelial, and cholangiocarcinomas.

**Conclusions:**

Our findings suggest that MGP is a newly identified sensitive IHC marker to support breast origin. MGP, TRPS1, and GATA3 could be applied as a reliable diagnostic panel to determine breast origin in clinical practice.

**Supplementary Information:**

The online version contains supplementary material available at 10.1186/s13058-022-01569-1.

## Introduction

Breast cancer is the most common cancer among women worldwide. Approximately 5.8% of female breast cancers have distant metastasis at diagnosis (de novo stage IV breast cancer) [1]. The annual prevalence of recurrent metastatic breast cancer (initially diagnosed with stage I–III breast cancer and later progresses to stage IV breast cancer) is approximately three times that of de novo stage IV breast cancer [2]. Considering the high prevalence and high rate of metastasis of breast cancer, metastatic breast carcinoma is commonly considered during differential diagnosis when metastatic disease is detected in lymph nodes or organs such as the lung, liver, bone, and brain in females [3, 4]. In addition to the tumor morphology and documented clinical history, sensitive and specific immunohistochemical (IHC) markers are helpful to determine the breast origin.

Currently, GATA-binding protein 3 (GATA3), gross cystic disease fluid protein 15 (GCDFP-15), and mammaglobin are commonly used breast cancer-specific IHC markers in clinical practice [5]. GATA3 is the most widely used breast-specific marker and has an overall sensitivity of > 90% [6–8]. GCDFP-15 and mammaglobin show lower sensitivities with high interstudy variation, which range from 40 to 75% and 40 to 70% [9–14], respectively. However, all these markers have been reported to show lower sensitivities in ER-negative breast cancer, especially in triple-negative breast cancer (TNBC), with sensitivities of GATA3 at 40–70% and GCDFP-15 and mammaglobin at < 30% [15–18]. Thus, there is a need to identify novel sensitive and specific markers and IHC panels for breast carcinomas.

In the present study, we first identified six potential genes that were specifically upregulated in breast carcinoma, namely N-acetyltransferase 1 (NAT1), matrix Gla protein (MGP), secretoglobin family 2A member 2 (SCGB2A2/mammaglobin-A), LIM homeobox transcription factor 1 beta (LMX1B), transcription factor AP-2 beta (TFAP2B), and transcriptional repressor GATA binding 1 (TRPS1), by bioinformatics analyses using The Cancer Genome Atlas (TCGA) and Clinical Proteomic Tumor Analysis Consortium (CPTAC) databases across 24 different solid tumors. We further observed that only TRPS1 and matrix Gla protein (MGP) had comparable expression in luminal A/B, HER2-enriched, basal-like and normal-like subtypes of breast cancer. We next compared MGP, TRPS1, and GATA3 expression in 1201 breast carcinoma cases of different subtypes, including 140 metaplastic breast carcinoma cases, using immunochemistry staining. Moreover, as a newly identified marker to support breast origin, MGP expression was also evaluated in solid tumors (*n* = 2221) and normal tissues (*n* = 1351) from different organs.

## Materials and methods

### Differential expression analysis in TCGA database

TCGA mRNA expression data (transcripts per million [TPM] and raw count) were downloaded from the Gene Expression Omnibus (GEO) database under the accession number GSE62944 [19]. TCGA breast cancer PAM50 subtypes were obtained from Du et al. [20]. ER, PR, and HER2 status for TCGA breast cancer was obtained from Thennavan et al. [21]. Differential expression (DE) analysis was performed with the R package DESeq2 (version 1.30.0) [22], and genes with fold change > 4 and false discovery rate (FDR) < 0.05 were selected as upregulated DE genes. Overlapping upregulated DE genes between breast carcinoma (BRCA) and 23 other tumor types (ACC, BLCA, CESC, COAD, DLBC, GBM, HNSC, KICH, KIRC, KIRP, LAML, LGG, LIHC, LUAD, LUSC, OV, PRAD, READ, SKCM, STAD, THCA, UCEC, UCS, Additional file 2: Table S1) were defined as breast-specific genes. Genes with low expression (median TPM < 1) were further filtered.

### Differential expression analysis in paired breast cancer metastases

mRNA expression data of 54 matched pairs of primary breast cancers and their metastases (brain, *n* = 22; bone, *n* = 11; ovary, *n* = 14; GI tract, *n* = 7) were downloaded from https://github.com/leeoesterreich/shiny-server/tree/master/apps/Paired_Mets [23–25]. The R package Limma (version 3.46.0) [26] was used to perform DE analysis with paired samples. Genes were defined as significantly downregulated in breast cancer metastasis compared to primary breast cancer if a fold change < 1 and FDR < 0.05 were found between all paired samples of primary breast cancers and breast metastases or between paired primary breast cancers and metastases from any specific site (brain, bone, ovary, or GI tract).

### Protein and mRNA correlation analysis

Normalized protein expression data of 105 TCGA breast cancer samples were downloaded from the CPTAC (https://cptac-data-portal.georgetown.edu/study-summary/S015) [27]. Spearman’s correlation between protein expression levels and mRNA expression levels (TPM) was performed using R v.4.0.3.

### Human tumor and normal tissue samples

Tissue microarrays (TMAs) of breast carcinoma (invasive breast carcinoma of no special type, *n* = 1061), hepatocellular carcinoma (*n* = 318), ovarian carcinoma (*n* = 290), cholangiocarcinoma (*n* = 163), renal cell carcinoma (*n* = 121), lung adenocarcinoma (*n* = 297), colorectal adenocarcinoma (*n* = 346), gastric adenocarcinoma (*n* = 198), urothelial carcinoma (*n* = 218), thyroid carcinoma (*n* = 144), melanoma (*n* = 126), as well as normal tissues of liver (*n* = 159), ovary (*n* = 121), biliary duct (*n* = 159), kidney (*n* = 61), lung (*n* = 212), colorectum (*n* = 206), stomach (*n* = 137), bladder (*n* = 152), and thyroid (*n* = 144) were made in the Department of Pathology at the Sun Yat-sen University Cancer Center (SYSUCC). In addition, 140 formalin-fixed paraffin-embedded (FFPE) tissues from 129 patients with metaplastic breast carcinoma (MBC) were also included. All cases were previously diagnosed at SYSUCC during the year 2015–2020. For invasive breast carcinoma of no special type (NST) cases, the representative areas of both tumor and non-tumor normal tissues for each case were selected with 2:1 ratio and circled to match the blocks for the TMA analysis. For other tumor cases, two representative tumor areas for each case were selected for the TMA analysis. In addition, normal tissues from liver (*n* = 159), kidney (*n* = 61), ovary (*n* = 121), biliary duct (*n* = 159), lung (*n* = 212), colorectum (*n* = 206), stomach (*n* = 137), bladder (*n* = 152), and thyroid (*n* = 144) were also selected for the TMA analysis. This study was conducted in accordance with ethical standards and approved by the Ethics Committee of SYSUCC.

Estrogen receptor (ER), progesterone receptor (PR), and human epidermal growth factor receptor 2 (HER2) status of breast carcinoma were routinely evaluated according to The American Society of Clinical Oncology/College of American Pathologists guideline recommendations [28, 29]. Herein, breast carcinomas were categorized into three groups based on ER, PR, and HER2 status as follows: ER and PR positive with HER2 negative (ER/PR+ group); HER2 positive regardless of ER and PR status (HER2+ group); and ER, PR, and HER2 negative (TNBC group).

### IHC analysis

Tissue microarrays and whole-tissue slides were stained with antibodies against MGP (mouse monoclonal, A-11; Santa Cruz Biotechnology, Dallas, TX, USA; dilution 1:100), TRPS1 (rabbit monoclonal, EPR16171; Abcam, Cambridge, MA, USA; dilution 1:800), and GATA3 (mouse monoclonal, EP368; Gene Tech, Shanghai, CHINA; dilution 1:600). Normal breast tissue samples were used as positive (incubated with primary antibody) and negative (incubated with antibody diluent) controls.

**Fig. 1:**
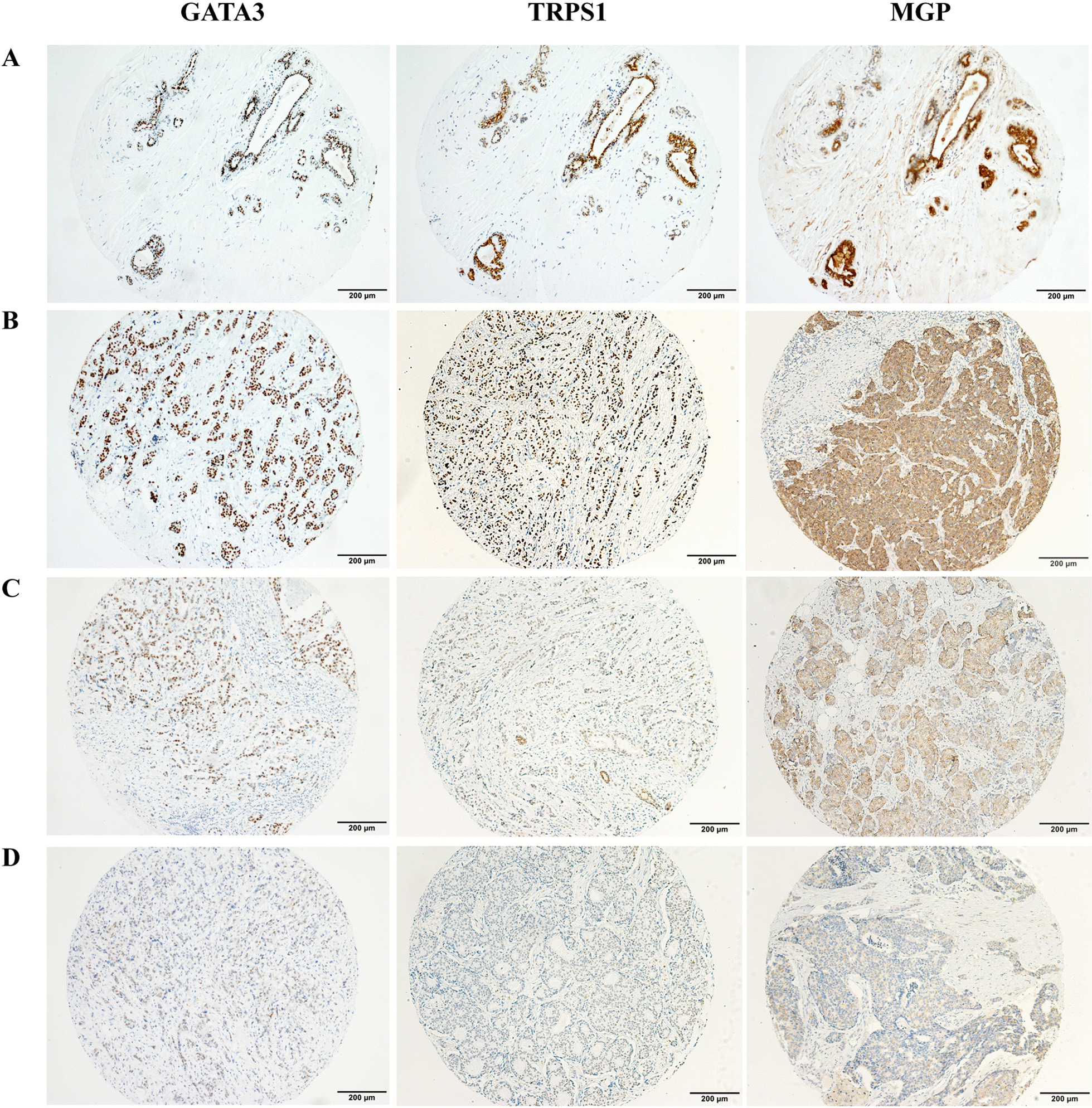
GATA3, TRPS1, and MGP expression in benign breast ducts and breast cancer. GATA3 and TRPS1 showed nuclear staining and MGP showed cytoplasmic staining in the benign ductal epithelial cells (**A**). Representative immunostaining images showing high (**B**), moderate (**C**), and mild (**D**) expression of GATA3, TRPS1, and MGP

MGP, TRPS1, and GATA3 IHC stains were reviewed and scored by pathologists PS, JH, XC, and ML. For MGP, cytoplasmic staining was considered positive. For TRPS1 and GATA3, only nuclear staining was considered positive. Immunoreactive scores were obtained by multiplying the percentage score (0, < 1%; 1, 1–10%; 2, 11–50%; 3, 51–100% cells positive) by the staining intensity (0, negative; 1, weak; 2, moderate; 3, strong) [30]. The degrees of MGP, TRPS1, and GATA3 expression were categorized as negative (0–1), mild positive (2), moderate positive (3–4), or high positive (6 and 9) based on immunoreactive scores (Fig. 1). Every case was reviewed and scored by two pathologists independently. Cases showing discrepant categorized results by the two pathologists were determined by discussion, and agreement was made by at least three pathologists.

### Statistical analysis

Statistical analysis was performed using R (version 4.0.3). Categorical variables are presented as counts/total (percentage). The chi-square test of independence or two-sided Fisher exact test (if any cell of the 2 × 2 table had an expected count less than 5) was used to test whether two categorical variables (IHC positivity and gene) are related to each other. Post hoc Bonferroni correction was performed for multiple comparisons. A *p* value less than 0.05 was considered statistically significant.

## Results

### Identification of potential breast-specific markers

**Fig. 2:**
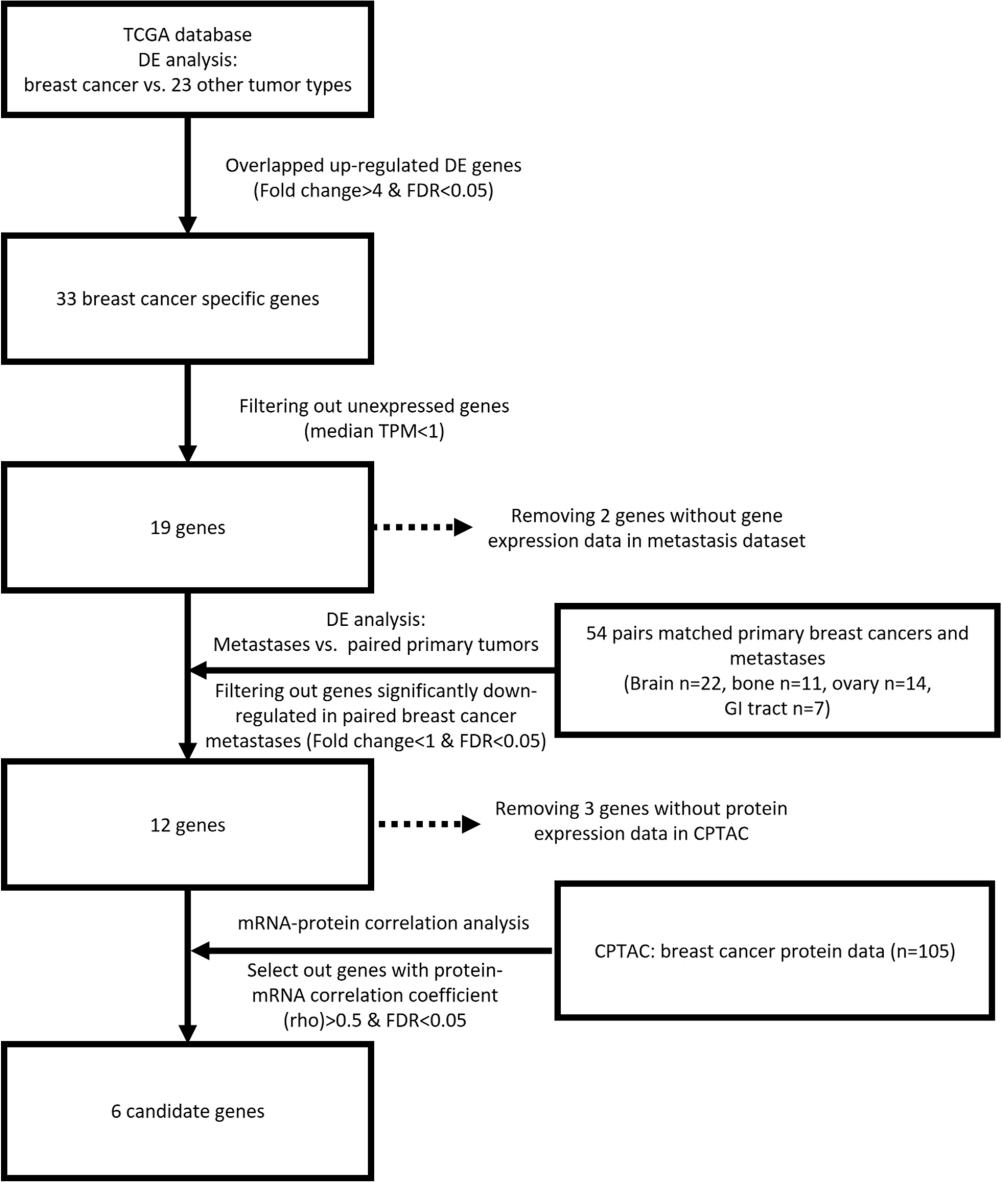
Schematic showing the selection of the breast-specific candidate markers using bioinformatic approach

A flowchart showing the identification of breast-specific candidate markers using a bioinformatic approach is shown in Fig. 2. We first performed differential gene expression analysis between breast carcinoma and 23 other tumor types (Additional file 2: Table S1). Thirty-three genes were commonly upregulated in breast carcinoma compared to other tumors, of which 19 genes had a median transcript per million (TPM) > 1. To identify genes highly expressed in both primary breast carcinoma and its metastasis, we removed genes that were significantly downregulated in metastatic breast carcinoma (Additional file 3: Table S2). Among the 12 remaining genes, six genes had a high correlation (correlation coefficient > 0.5, FDR < 0.05) between protein and mRNA expression levels (Additional file 4: Table S3), including LMX1B, TRPS1, NAT1, MGP, SCGB2A2, and TFAP2B, which could serve as potential IHC markers to support breast origin.

### MGP, TRPS1, and GATA3 expression in breast carcinomas

**Fig. 3:**
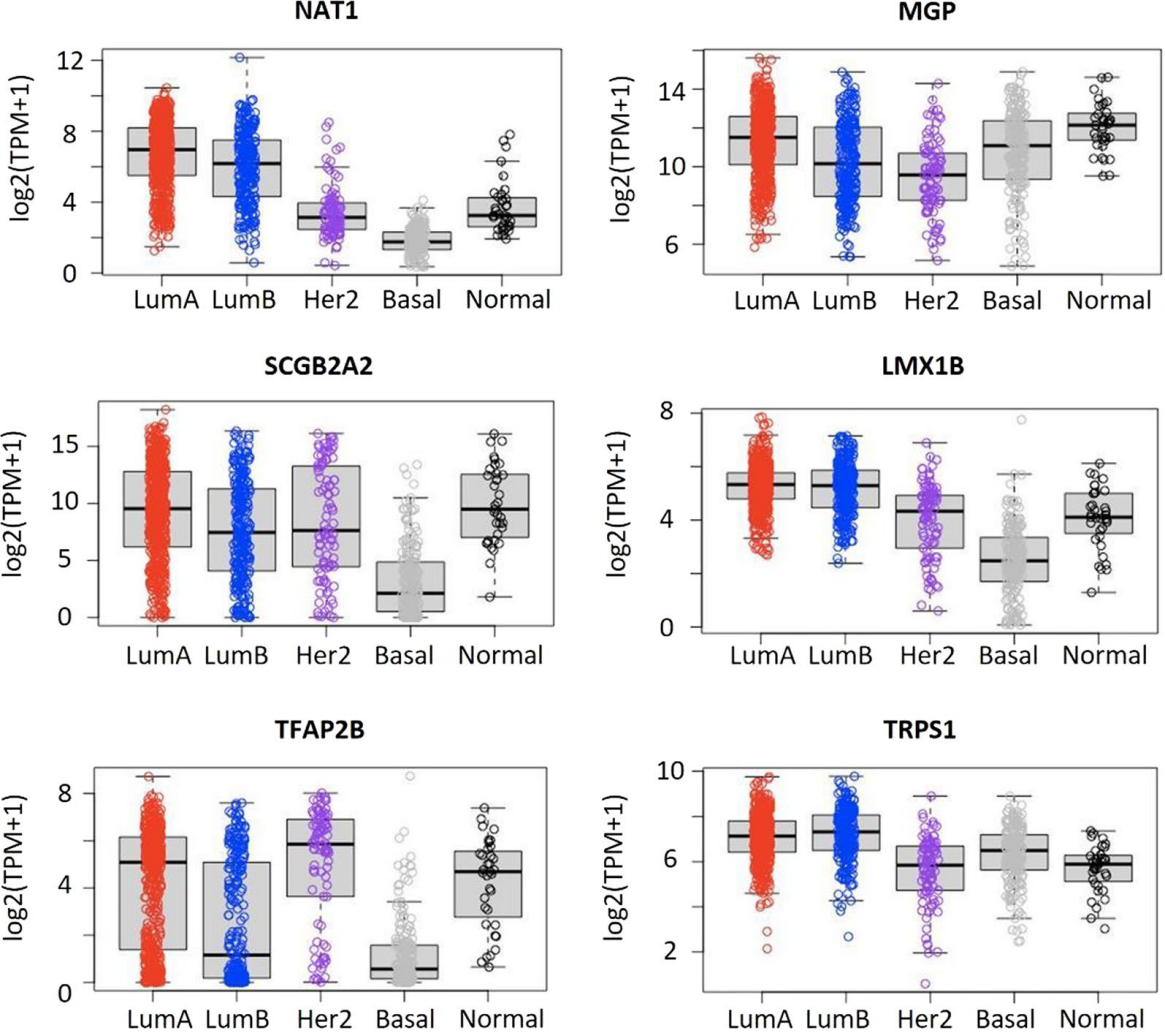
The mRNA levels of candidate genes in different subtypes of breast cancer. LumA, luminal A; LumB, luminal B; Her2, Her2-enriched; Basal, basal-like; Normal, normal-like

Since MGP and TRPS1 showed high expression in luminal A/B, HER2-enriched, TNBC/basal-like, and normal-like subtypes according to the PAM50 subtyping (Fig. 3, Additional file 1: Figure S1), we selected MGP, TRPS1, and GATA3 for further validation in 1201 cases of breast carcinoma of different subtypes and normal breast tissues.

As shown in Fig. 1, GATA3 and TRPS1 showed nuclear staining while MGP showed cytoplasmic staining in the benign ductal epithelial cells, but not in myoepithelial cells. Immunoreactive scores of the enrolled cases are shown in Additional file 5: Table S4. MGP, TRPS1, and GATA3 expression in breast carcinomas is summarized in Table 1. MGP, TRPS1, and GATA3 were positive in 1075/1201 (89.5%), 1109/1201 (92.3%), and 922/1201 (76.8%) breast carcinomas, respectively. Comparable MGP and TRPS1 positivity was observed in HER2-positive (91.2% vs. 92.0%, *p* = 0.79) and TNBC subtypes (87.3% vs. 91.2%, *p* = 0.18), while MGP had relative lower positivity in ER/PR+ subtype (89.6% vs. 93.1%, *p* < 0.05). Although 93.4% of ER/PR+ tumors showed GATA3 expression, the positivity rates of GATA3 were lower than those of MGP or TRPS1 in HER2+ (77.0%, adjusted *p* < 0.001 for GATA3 vs. MGP and GATA3 vs TRPS1) and TNBC (43.3%, adjusted *p* < 0.001 for GATA3 vs. MGP and GATA3 vs TRPS1) subtypes. In ER/PR-positive tumors, most cases showed moderate–high expression of GATA3, which was more than those in TRPS1 or MGP (88.8% vs. 80.4% vs. 57.4%, adjusted *p* < 0.001 for GATA3 vs. MGP and GATA3 vs TRPS1). In contrast, the moderate–high positivity rate of GATA3 was significantly lower than that of TRPS1 or MGP in TNBC subtypes (30.3% vs. 80.6% vs. 68.0%, adjusted *p* < 0.001 for GATA3 vs. MGP and GATA3 vs TRPS1).

**Table 1:** GATA3, TRPS1, and MGP expression in breast carcinomas

	Subtype	Negative (*n*/%)	Positive (*n*/%)			Total (*n*)
			Mild	Moderate	High	
GATA3	ER/PR+	37 (6.6)	26 (4.6)	73 (12.9)	429 (75.9)	565
	HER2+	81 (23.0)	33 (9.4)	64 (18.2)	174 (49.4)	352
	TNBC-NSTs	87 (60.4)	7 (4.9)	8 (5.6)	42 (29.2)	144
	TNBC-MBCs	74 (52.9)	30 (21.4)	21 (6.7)	15 (10.7)	140
	Total	279 (23.2)	96 (8.0)	166 (13.8)	660 (55.0)	1201
MGP	ER/PR+	59 (10.4)	153 (27.1)	163 (28.8)	190 (33.6)	565
	HER2+	31 (8.8)	78 (22.2)	103 (29.3)	140 (39.8)	352
	TNBC-NSTs	20 (13.9)	30 (20.8)	44 (30.6)	50 (34.7)	144
	TNBC-MBCs	16 (11.4)	25 (17.9)	49 (35.0)	50 (35.7)	140
	Total	126 (10.5)	286 (23.8)	359 (29.9)	430 (35.8)	1201
TRPS1	ER/PR+	39 (6.9)	72 (12.7)	141 (25.0)	313 (55.4)	565
	HER2+	28 (8.0)	55 (15.6)	92 (26.1)	177 (50.3)	352
	TNBC-NSTs	22 (15.3)	22 (15.3)	36 (25.0)	64 (44.4)	144
	TNBC-MBCs	3 (2.1)	8 (5.7)	17 (12.1)	112 (80.0)	140
	Total	92 (7.7)	157 (13.1)	286 (23.8)	666 (55.4)	1201

ER, estrogen receptor; PR, progesterone receptor; HER2, human epidermal growth factor receptor-2; TNBC, triple-negative breast cancer; NST, no special type; MBC, metaplastic breast carcinoma

### MGP, TRPS1, and GATA3 expression in metaplastic breast carcinomas

As shown in Table 1, MGP, TRPS1, and GATA3 were positive in 124/140 (88.6%), 137/140 (97.9%), and 66/140 (47.1%) TNBC-MBC patients, respectively. TNBC-MBC showed comparable positive staining of MGP (88.6% vs. 86.1%, *p* = 0.66) or GATA3 (47.1% vs. 39.6%, *p* = 0.24) than in TNBC of no special type (TNBC-NST) group. Notably, the positivity rate of TRPS1 was significantly higher in TNBC-MBCs than in TNBC-NSTs (97.9% vs. 84.7%, *p* < 0.001). According to histological subtypes, as shown in Table 2, MGP, GATA3, and TRPS1 were found to be positive in 86.5% (64/74), 62.2% (46/74), and 98.6% (73/74) of squamous cell carcinomas (SqCC), 83.3% (15/18), 22.2% (4/18), and 94.4% (17/18) of spindle cell carcinomas (SpCC), 92.5% (37/40), 32.5% (13/40), and 100% (40/40) of metaplastic breast carcinomas with mesenchymal differentiation (MBC-MD), and 100% (8/8), 37.5% (3/8), and 87.5% (7/8) of fibromatosis-like metaplastic carcinomas (FMC).

**Table 2:** GATA3, TRPS1, and MGP expression in metaplastic breast carcinomas

	GATA3				MGP				TRPS1			
	Negative (*n*/%)	Positive (*n*/%)			Negative (*n*/%)	Positive (*n*/%)			Negative (*n*/%)	Positive (*n*/%)		
		Mild	Moderate	High		Mild	Moderate	High		Mild	Moderate	High
SqCC (*n* = 74)	28 (37.8)	16 (21.6)	16 (21.6)	14 (18.9)	10 (13.5)	18 (24.3)	22 (29.7)	24 (32.4)	1 (1.4)	4 (5.4)	10 (13.5)	59 (79.7)
SpCC (*n* = 18)	14 (77.8)	1 (5.6)	2 (11.1)	1 (5.6)	3 (16.7)	1 (5.6)	10 (55.6)	4 (22.2)	1 (5.6)	2 (11.1)	0 (0)	15 (83.3)
MBC-MD (*n* = 40)	27 (67.5)	10 (25.0)	3 (7.5)	0 (0)	3 (7.5)	4 (10.0)	13 (32.5)	20 (50.0)	0 (0)	1 (2.5)	4 (10.0)	35 (87.5)
FMC (*n* = 8)	5 (62.5)	3 (37.5)	0 (0)	0 (0)	0 (0)	2 (25.0)	4 (50.0)	2 (25.0)	1 (12.5)	1 (12.5)	3 (37.5)	3 (37.5)

SqCC, squamous cell carcinoma; SpCC, spindle cell carcinoma; MBC-MD, metaplastic breast carcinoma with mesenchymal differentiation; FMC, fibromatosis-like metaplastic carcinoma

### Validation of the IHC panel of MGP, GATA3, and TRPS1

**Fig. 4:**
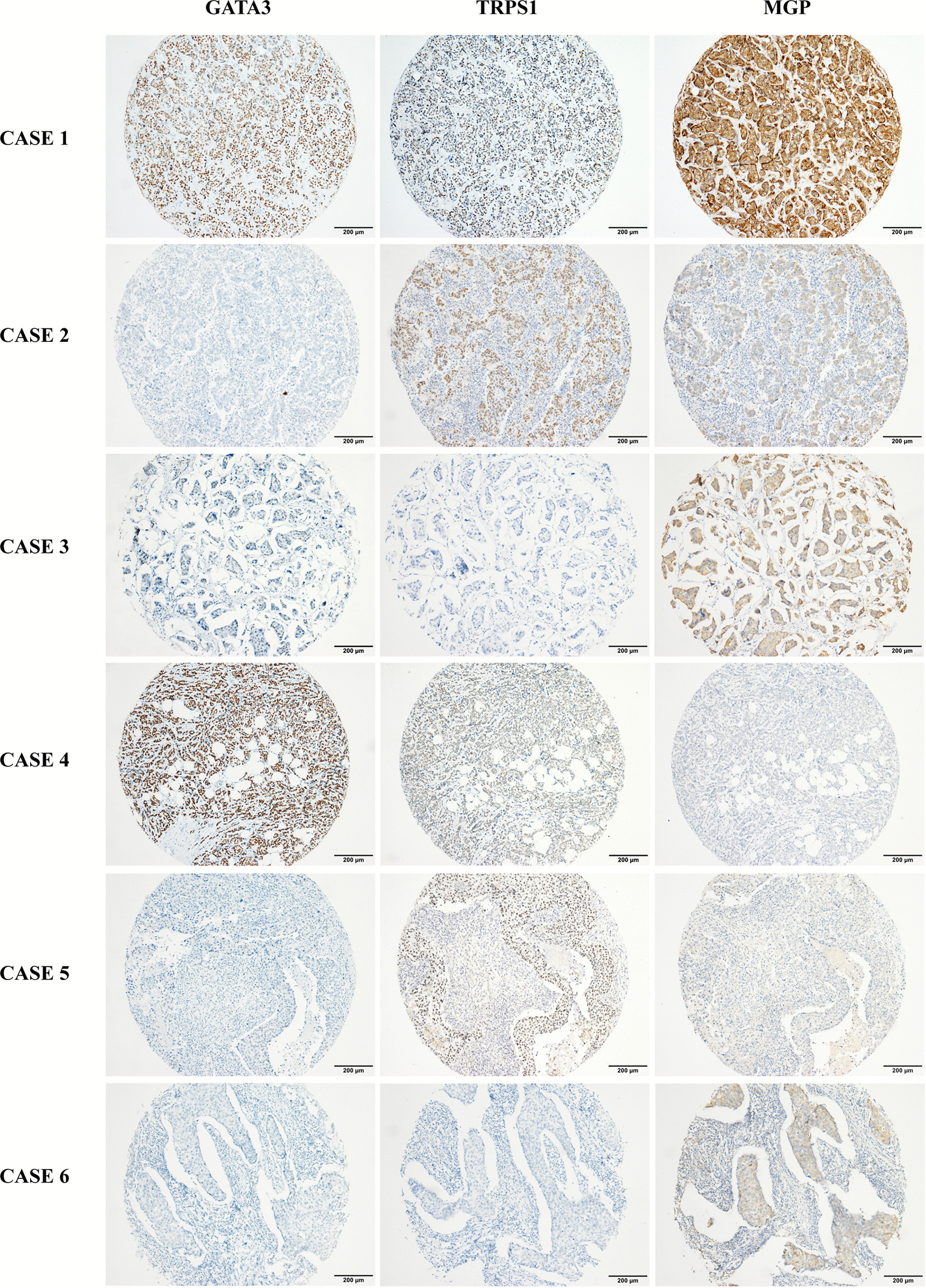
Representative cases showing the combined positive using GATA3, TRPS1, and MGP panel in breast cancer. Case 1 shows an ER/PR+ invasive breast carcinoma with high expression of GATA3, TRPS1, and MGP. Case 2 shows a triple-negative invasive breast carcinoma with high expression of TRPS1, moderate expression of MGP, and negative expression of GATA3. Case 3 shows a HER2-positive (3+) invasive micropapillary carcinoma with high expression of MGP, mild expression of GATA3, and negative expression of TRPS1. Case 4 shows an ER/PR+ invasive breast carcinoma with high expression of GATA3, moderate expression of TRPS1, and negative expression of MGP. Case 5 shows a triple-negative invasive breast carcinoma with high expression of TRPS1, but negative expression of GATA3 and MGP. Case 6 shows a triple-negative invasive breast carcinoma with moderate expression of MGP, but negative expression of GATA3 and TRPS1

**Fig. 5:**
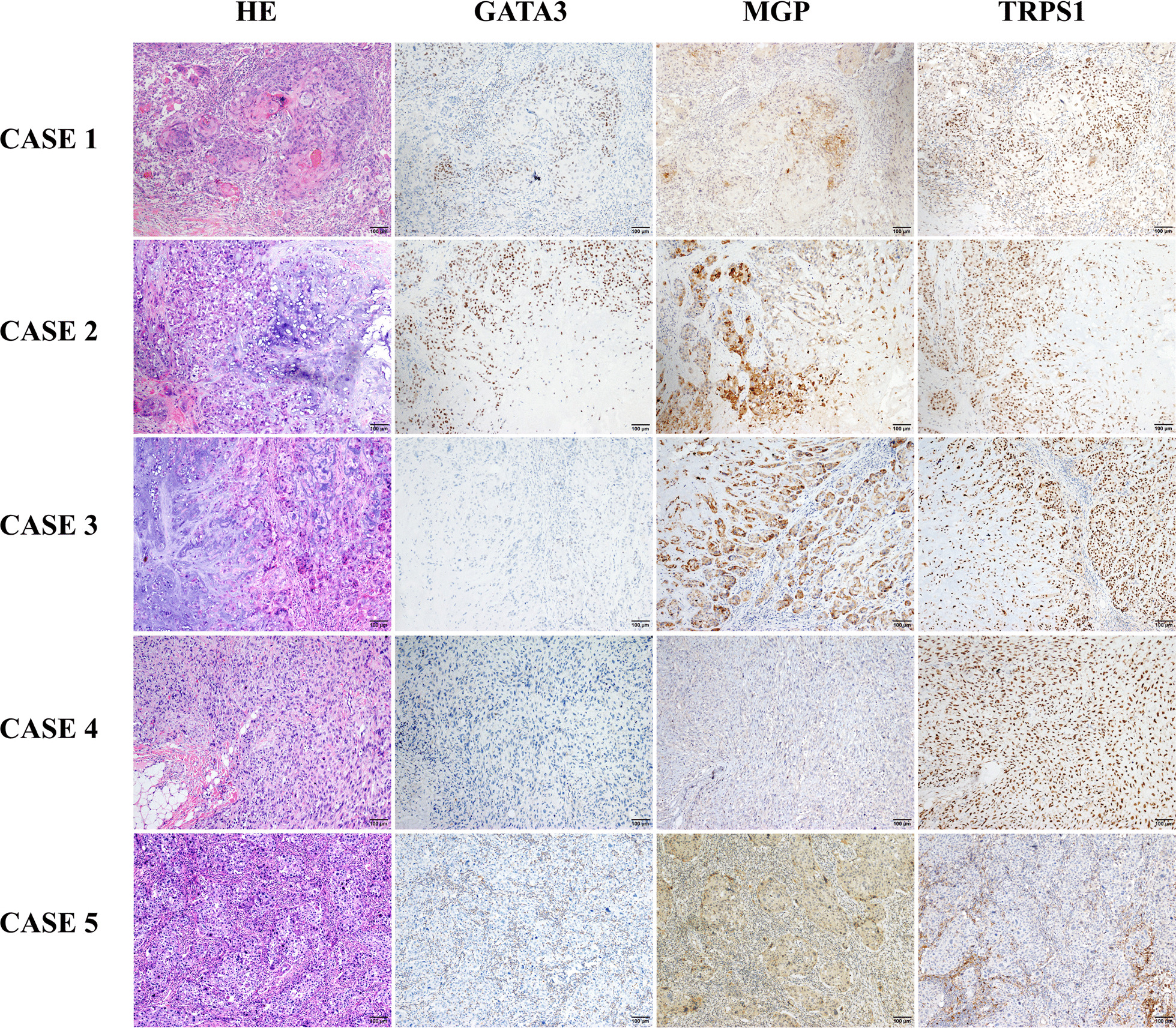
Representative cases showing the combined positive using GATA3, TRPS1, and MGP panel in metaplastic breast carcinomas. Case 1 shows a metaplastic squamous cell carcinoma with high expression of GATA3, MGP, and TRPS1. Case 2 shows a metaplastic breast carcinoma with chondrogenic differentiation and high expression of GATA3, MGP, and TRPS1. Case 3 shows a metaplastic breast carcinoma with chondrogenic differentiation which exhibits high expression of MGP and TRPS1, but negative for GATA3. Case 4 shows a high-grade spindle cell carcinoma with high expression of TRPS1, and negative expression of GATA3 and MGP. Case 5 shows a metaplastic squamous cell carcinoma with moderate expression of MGP, but negative expression of GATA3 and TRPS1

When combining GATA3, TRPS1, and MGP, we observed that 797/1201 (66.4%) of the enrolled breast carcinoma cases were positive for all three markers, including 452/565 (80.0%), 238/352 (67.6%), and 107/284 (37.7%) in the ER/PR+, HER2+, and TNBC subgroups, respectively (Table 3, Figs. 4 and 5). Among all enrolled cases, 36/1201 (3.0%; 7 ER/PR+, 13 HER2+, 16 TNBC) were positive for MGP, while TRPS1 and GATA3 were both negative; 40/1201 (3.3%; 10 ER/PR+, 7 HER2+, 23 TNBC) were positive for TRPS1, while MGP and GATA3 were both negative; 8/1201 (0.7%; 7 ER/PR+, 1 TNBC) were positive for GATA3, while MGP and TRPS1 were both negative. Only 9 cases (0.7%; 2 ER/PR+, 2 HER2+, 5 TNBC) were negative for all three markers.

**Table 3:** Combined positive rates of GATA3, MGP, and TRPS1 in breast carcinomas

IHC panel	Subtype	Diagnostic criteria*		
		At least one positive (*n*/%)	At least two positive (*n*/%)	All three positive (*n*/%)
GATA3, MGP	ER/PR+	556 (98.4)	478 (84.6)	–
	HER2+	344 (97.7)	248 (70.5)	–
	TNBC-NSTs	131 (91.0)	50 (34.7)	–
	TNBC-MBCs	130 (92.9)	60 (42.9)	–
	Total	1161 (96.7)	836 (69.6)	–
GATA3, TRPS1	ER/PR+	558 (98.8)	496 (87.8)	–
	HER2+	335 (95.2)	260 (73.9)	–
	TNBC-NSTs	125 (86.8)	54 (37.5)	–
	TNBC-MBCs	138 (98.6)	65 (46.4)	–
	Total	1156 (96.3)	875 (72.9)	–
MGP, TRPS1	ER/PR+	557 (98.6)	475 (84.1)	–
	HER2+	349 (99.1)	296 (84.1)	–
	TNBC-NSTs	139 (96.5)	107 (74.3)	–
	TNBC-MBCs	139 (99.3)	122 (87.1)	–
	Total	1184 (98.6)	1000 (83.3)	–
GATA3, MGP, TRPS1	ER/PR+	563 (99.6)	545 (96.5)	452 (80.0)
	HER2+	350 (99.4)	328 (93.2)	238 (67.6)
	TNBC-NSTs	139 (96.5)	117 (81.3)	47 (32.6)
	TNBC-MBCs	140 (100.0)	127 (90.7)	60 (42.9)
	Total	1192 (99.3)	1117 (93.0)	797 (66.4)

IHC, immunohistochemical staining; ER, estrogen receptor; PR, progesterone receptor; HER2, human epidermal growth factor receptor-2; TNBC, triple-negative breast cancer; NST, no special type; MBC, metaplastic breast carcinoma

*Immunoreactive scores indicating mild, moderate, and high expression were all counted as positive

Different composite uses of MGP, TRPS1, and GATA3 may bring in GATA3-MGP, GATA3-TRPS1, MGP-TRPS1, and GATA3-MGP-TRPS1 IHC panels in daily practice. Assuming that the criteria used to determine the breast origin require at least two markers positive in these IHC panels, we found that the GATA3-MGP-TRPS1 panel showed the highest sensitivity (90.7%) among all enrolled cases, followed by MGP-TRPS1 (83.3%), GATA3-TRPS1 (72.9%), and GATA3-MGP (69.6%). In the TNBC and HER2+ groups, the sensitivity of the GATA3-MGP-TRPS1 (TNBC, 85.9%; HER2+, 93.2%) and MGP-TRPS1 (TNBC, 80.6%; HER2+, 84.1%) panels was significantly higher (adjusted *p* < 0.01 for all comparisons) than that of GATA3-MGP (TNBC, 38.7%; HER2+, 70.5%) and GATA3-TRPS1 (TNBC, 41.9%; HER2+, 73.9%). In ER/PR+ tumors, GATA3-MGP-TRPS1 demonstrated the best performance (96.5%, adjusted *p* < 0.001 for all comparisons), while the other three panels had comparable sensitivity at 84.1–87.8% (adjusted *p* > 0.05 for all comparisons); see Table 3 for more details.

### MGP expression in other solid tumors and normal tissues

**Fig. 6:**
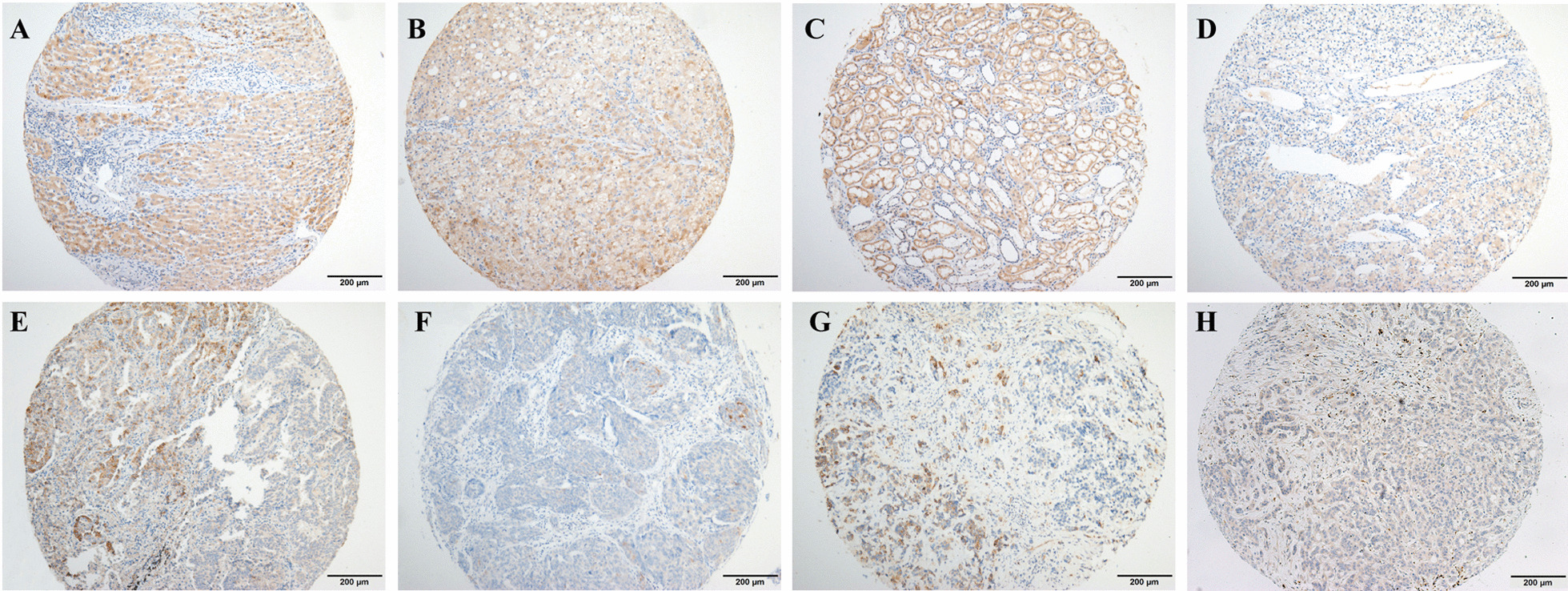
MGP expression in other solid tumors and normal tissues. MGP showed constant moderate expression in normal hepatocytes (**A**) and renal tubules (**C**). Mild-to-moderate expression of MGP was found in 31.1% of hepatocellular carcinoma (**B**). Rare cases (0.6–5%) had focal MGP expression in renal cell carcinoma (**D**), ovarian carcinoma (**E**), lung adenocarcinoma (**F**), urothelial carcinoma (**G**), and cholangiocarcinoma (**H**)

Since the specificity of TRPS1 in breast carcinomas has been recently reported by Ai et al. [31], herein, as a newly identified marker to support breast origin, MGP expression was further evaluated in solid tumors (*n* = 2384) and normal tissues (*n* = 1351) from different organs (Table 4). MGP was negative in colorectal adenocarcinoma, gastric adenocarcinoma, thyroid carcinoma, and melanoma, while mild-to-moderate positivity was found in 31.1% (99/318) of hepatocellular carcinomas. Individual cases had focal MGP expression in renal cell carcinoma (6/121, 5.0%), ovarian carcinoma (7/290, 2.4%), lung adenocarcinoma (2/297, 0.7%), urothelial carcinoma (2/218, 0.9%) and cholangiocarcinoma (1/163, 0.6%), as shown in Fig. 6. In normal tissues, we observed that MGP showed mild-to-moderate positive expression in normal hepatocytes (159/159) and renal tubules (61/61) but the negative expression in other organs, including the ovary, biliary duct, lung, colorectum, stomach, bladder, and thyroid.

**Table 4:** MGP expression in other solid tumors and normal tissues

	Type	MGP-negative	MGP-positive	Total (*n*)
Tumors	Breast carcinoma	126 (10.5)	1075 (89.5)	1201
	Hepatocellular carcinoma	219 (68.9)	99 (31.1)	318
	Ovarian carcinoma	283 (97.6)	7 (2.4)	290
	Renal cell carcinoma	114 (94.2)	6 (5.0)	121
	Lung adenocarcinoma	295 (99.3)	2 (0.7)	297
	Urothelial carcinoma	216 (99.1)	2 (0.9)	218
	Cholangiocarcinoma	162 (99.4)	1 (0.6)	163
	Colorectal adenocarcinoma	346 (100)	0 (0)	346
	Gastric adenocarcinoma	198 (100)	0 (0)	198
	Thyroid carcinoma	144 (100)	0 (0)	144
	Melanoma	126 (100)	0 (0)	126
Normal tissues	Liver (hepatocyte)	0 (0)	159 (100)	159
	Kidney (renal tubule)	0 (0)	61 (100)	61
	Ovary	121 (100)	0 (0)	121
	Biliary duct	159 (100)	0 (0)	159
	Lung	212 (100)	0 (0)	212
	Colorectum	206 (100)	0 (0)	206
	Stomach	137 (100)	0 (0)	137
	Bladder	152 (100)	0 (0)	152
	Thyroid	144 (100)	0 (0)	144

## Discussion

Specific IHC markers supporting the breast origin of an unknown carcinoma are important and helpful for diagnosis, especially ER-negative or triple-negative tumors. Currently, GATA3, GCDFP-15, and mammaglobin are commonly used panels to support breast origin, of which GATA3 is the most widely used. GATA-binding protein 3 (GATA3) is considered to be the most prevalent transcription factor involved in the proliferation and differentiation of ductal epithelium of the breast [32–34], which is linked to ER signaling [35, 36]. A number of studies have demonstrated that GATA3 is a superior marker for ER+ breast carcinoma than GCDFP-15 or mammaglobin, with a sensitivity consistently over 90% [7, 16, 35]. However, the sensitivity of GATA3 is significantly lower in ER-negative or TNBC subtypes, ranging from 15 to 60% in various studies [7, 16, 31, 35, 37, 38]. Our data also show that GATA3 exhibited extremely high sensitivity in 93.4% of ER/PR+ breast carcinomas, while up to 23.0% HER2 + BC and 60.4% TNBC were negative for GATA3. In addition, GATA3 is not a breast-specific marker that can label other common sources of tumors [39], including urothelial carcinomas, squamous cell carcinomas, lung adenocarcinoma, pancreatic adenocarcinomas, endocrine tumors, soft tissue sarcomas, and others. Currently, no single IHC marker is entirely breast-specific; GATA3 should be applied as part of an IHC panel, and more specific biomarkers are still required in the diagnostic setting.

Using mRNA sequencing and proteomic data from TCGA and CPTAC of 24 different solid tumors, we identified six potential genes that are specifically upregulated in breast carcinoma: NAT1, MGP, SCGB2A2, LMX1B, TFAP2B, and TRPS1. Similar to the approach used by Ai et al. [29], the genes highly expressed in breast carcinoma compared to all other tumor types and equally highly expressed in all four PAM50 subtypes were identified as candidate markers. Moreover, our approach includes two more steps. Since gene expression may change during the process of metastasis, we also removed genes showing decreased expression in metastasis using RNA-Seq data from primary breast lesions and their paired metastases. In addition, we aimed to look for potential biomarkers that can be used in IHC-based assays, which are cellular protein labeling techniques; thus, we further selected genes with a high correlation between protein and RNA expression using protein expression data from CPTAC. SCGB2A2 (mammaglobin) and the most recently reported breast-specific marker TRPS1 are both listed in our final candidate markers, which suggests the robustness of our approach. This bioinformatic analysis process (Fig. 2) not only identifies MGP and TRPS1 as novel candidate IHC markers to support breast origin but also provides a new approach for the future selection of specific biomarkers in other tumor types.

Trichorhinophalangeal syndrome type 1 (TRPS1) is named for a very rare hereditary disease with mainly autosomal dominant inheritance features characterized by craniofacial and skeletal abnormalities with damage and mutation affecting chromosome 8q [40]. TRPS1 is reported to be a transcriptional repressor that binds specifically to GATA sequences and represses the expression of GATA-regulated genes which function in vertebrate development, especially in the process of chondrocyte proliferation and differentiation [41, 42]. Some studies have suggested that TRPS1 may also act as a critical modulator in mammary epithelial cell growth, differentiation, and breast cancer development via epithelial–mesenchymal transformation, DNA replication, and mitosis [43, 44]. A recent study by Ai et al. reported that TRPS1 could serve as a sensitive and specific marker for breast carcinomas [31]. TRPS1 exhibited high sensitivity in ER/PR+ (98%), HER2+ (87%), and TNBC (86%) subtypes on TMAs. On the other hand, TRPS1 showed no or little expression in other tumor types. Parkinson et al. [37] and Yoon et al. [38] further verified the utility of TRPS1, showing higher sensitivity in the HER2+ and TNBC subgroups. Although the commercial TRPS1 antibody used in our study (EPR16171 from Abcam) is different from Ai’s and Parkinson’s study (TRPS: PA5-845874 from Invitrogen/Thermo Fisher), similarly high TRPS1 expression (91.2–93.1%) is also found in all types of breast carcinoma with the largest sample size thus far. Our findings confirm that both clones of TRPS1 are sensitive markers supporting breast origin. In addition, previously reported abnormal membranous expression of TRPS1 was not observed in our cohort.

Previous studies reported that MGP is mainly secreted by chondrocytes [45, 46] and vascular smooth muscle cells [47], and it is considered a marker of vitamin K status in bone and vasculature, substantiating the role of MGP in extracellular matrix calcification regulation [48, 49]. MGP was recently found to be overexpressed in various types of cancer [50–52] and was reported to promote tumor progression by regulating angiogenesis [53]. In breast carcinoma, Yoshimura et al. and Gong et al. demonstrated that high MGP mRNA expression was associated with poor prognosis [52, 54]. However, whether MGP can serve as a breast-specific marker is unknown.

In our cohort of 1201 breast carcinomas, every case matched benign breast ducts in a separate TMA or whole-slide section. MGP displays cytoplasmic labeling in nearly all ductal epithelial cells with various strengths but not in myoepithelial cells. Perivascular smooth muscle can be used as an internal positive control for MGP (Fig. 1). MGP was verified as a reliable marker with extremely high sensitivity in all subtypes of breast carcinoma (87.3–91.2%), which is comparable to TRPS1 and much higher than GATA3 in HER2+ and TNBC subtypes.

We noticed that most MGP-positive cases demonstrated moderate and multifocal cytoplasmic staining patterns. There were generally more cases showing extensive and strong positivity for GATA3 and TRPS1 (greater than 49%) than for MGP (less than 40%, adjusted *p* < 0.001), except for the TNBC group (Table 1). Among the positive staining cases, 26.6% (286/1075, Table 1) showed mild positive of MGP, significantly higher than that of TRPS1 (14.1%, 157/1109, adjusted *p* < 0.001) and GATA3 (10.4%, 96/922, adjusted *p* < 0.001). More cases were categorized as mildly positive for MGP may be due to its cytoplasmic staining pattern, which is not preferable or easy to interpret subjectively like nuclear staining markers such as TRPS1 and GATA3. Mild cytoplasmic positivity tends to be more easily recognized as nonspecific or unstable as compared with mild nucleus positivity, which does affect the value of MGP as a single marker to determine breast origin in the clinical practice. Other commercial MGP antibodies could be also tested and verified in further studies. Even so, our data suggest that MGP has much better and more stable sensitivity than conventional nuclear (GATA3, SOX10 [38, 55, 56]) or cytoplasmic biomarkers (GCDFP15, mammaglobin) used to determine breast origin. The moderate–high positivity rate of MGP was significantly higher than that of GATA3 in TNBC-NST (65.3% vs. 34.8%, adjusted *p* < 0.001) and TNBC-MBC subtypes (70.7% vs. 17.4%, adjusted *p* < 0.001), suggesting the high sensitivity of MGP specially in the most troubling TNBCs. In addition, we observed that 239 GATA3-negative cases and 75 TRPS1-negative cases were positive for MGP, and 69 GATA3-mild positive cases and 96 TRPS1-mild positive cases showed moderate–high positive for MGP. Thus, using MGP, GATA3, and TRPS1 as a novel IHC panel significantly increased the sensitivity from 76.8–92.3% of the single marker (MGP, GATA3, or TRPS1) to 93.0–99.3% (≥ 1 positive or ≥ 2 markers positive for the GATA3, MGP, and TRPS1 panel).

Although our IHC data of MGP were collected from primary breast carcinomas, our bioinformatics analysis revealed that MGP mRNA was not significantly changed between paired primary tumors and their metastases (Additional file 3: Table S2), which suggests that similar MGP expression could be found in metastatic breast carcinomas. Further verification of MGP is required in metastatic lesions as well as special types of invasive breast carcinoma, such as salivary gland-type tumors and neuroendocrine carcinoma.

In the present study, we included 144 TNBC-NSTs and 140 TNBC-MBCs. GATA3 was expressed in only 39.6% of TNBC-NSTs and 47.1% of TNBC-MBCs and was mostly weakly positive, which is consistent with previous studies [31, 37, 38]. TRPS1 and MGP maintained high sensitivity in both TNBC-NSTs (84.7% and 86.1%) and TNBC-MBCs (97.9% and 88.6%). Focusing on MBCs, the sensitivity of TRPS1 (137/140, 97.9%) in our cohort was slightly higher than those reported by Ai et al. [31], Parkinson et al. [37], and Yoon et al. [38], which were 86.5% (45/52, adjusted *p* < 0.05), 91.0% (61/67, raw *p* = 0.061), and 95.0% (134/141, raw *p* = 0.33), respectively. TRPS1 exhibited a larger portion of strong positivity among the positive cases in TNBC-MBCs (112/137 [81.8%]) compared with the ER/PR+ (313/526 [59.5%], adjusted *p* < 0.001) or HER2+ (177/324 [54.6%], adjusted *p* < 0.001) group (Table 1). When MBCs were stratified by subtype, we observed that GATA3 showed relatively higher sensitivity in SqCCs (62.1%) than in other subtypes (22.5–37.5%). The majority of cases (85%, 34/40) in MBC with mesenchymal differentiation group showed chondroid/osseous differentiation, and both TRPS1 and MGP had the highest positivity in MBCs with chondroid/osseous differentiation (100% and 88.2%, respectively), followed by SqCCs and SpCCs. Interestingly, fibromatosis-like metaplastic carcinomas (FMCs) in our cohort were all positive for MGP (8/8). A total of 37.5% (3/8) and 87.5% (7/8) of FMCs were positive for TRPS1 and GATA3, respectively, which is inconsistent with Parkinson et al. [37] showing no single case with positive staining of TRPS1 or GATA3 in FMCs (0/3). In addition, we observed that 64 GATA3-negative MBCs and 2 TRPS1-negative MBCs were positive for MGP. The combined use of MGP, GATA3, and TRPS1 increased the sensitivity from 47.1–97.9% of the single marker (MGP, GATA3, or TRPS1) to 90.7–100.0% (≥ 1 positive or ≥ 2 markers positive for the GATA3, MGP, and TRPS1 panel) in MBCs. All these data suggest that both MGP and TRPS1 maintain excellent sensitivity in different subtypes of metaplastic breast carcinomas. However, TRPS1 may play a role in chondro-osseous differentiation. Wang et al. [57] found that TRPS1 was highly expressed in chondro-osseous sarcomas from both breast and extramammary sites, including heterologous components within malignant phyllodes tumors. Coincidentally, MGP is highly abundant in cartilage and acts as a critical regulator of calcification and turnover of bone and cartilage. The previous study showed that tumors exhibiting cartilaginous/osseous differentiation such as osteosarcoma and chondrosarcoma had high MGP expression [46, 58], and they can also metastasize to bone and lung-like breast cancer [59–62]. It would be hard to differentiate metaplastic breast carcinoma with cartilaginous/osseous differentiation from these tumors simply based on MGP positivity. Thus, pathologists should be cautious when faced with positive expression of MGP or TRPS1 in chondroid/osteoid components, especially with limited biopsy tissue. Further verification of MGP is also required in sarcomas and malignant phyllodes tumors.

MGP was first isolated from bovine bone matrix in the 1980s [43]; since then, its expression has been demonstrated in normal endothelial cells, fibroblasts, chondrocytes, and vascular smooth muscle cells. Our data show that MGP had negative expression in normal organs, including the ovary, biliary duct, lung, colorectum, stomach, bladder, and thyroid. Interestingly, we observed that MGP was constantly expressed in normal hepatocytes, but the positive expression was detected in only 31.1% of hepatocellular carcinomas. In addition, previous studies also demonstrated that MGP was abundantly expressed in normal kidneys, specifically in the epithelium of Bowman's capsule and proximal tubules, where the activated protein contributes to maintaining renal microvascular traits [63, 64]. Consistently, we found that MGP was predominantly expressed in normal renal tubules, but the positivity rate significantly dropped to 5.0% in renal cell carcinomas. It is difficult for MGP itself to differentiate breast carcinoma from hepatocellular or renal cell carcinoma. The joint application of TRPS1 and GATA3 may be helpful since TRPS1 [31, 37] and GATA3 [7, 65] have been proven to be rarely positive in these tumors.

Since high-grade ovarian serous carcinoma and breast carcinoma share similar morphologies and immunophenotypes, such as a micropapillary architecture and ER positivity, the diagnosis can be challenging. Our results showed that only 2.4% of ovarian serous carcinomas had focal MGP expression, which is lower than the reported positivity of GATA3 (~6%, [7, 66]) and TRPS1 (14%, [31]). Thus, this GATA3-MGP-TRPS1 panel may need inclusion with Pax-8 and WT-1 to differentiate breast carcinoma from serous carcinoma.

Poorly differentiated lung adenocarcinomas have been frequently reported as TTF-1-negative and occasionally labeled for ER [67], while individual cases of breast carcinoma may show TTF-1 staining [68]. Thus, differentiating breast carcinoma and lung adenocarcinoma is common and sometimes difficult in clinical practice. In the current study, MGP was rarely expressed in lung adenocarcinomas (0.7%), which is lower than the previously reported positivity of GATA3 (∽8%, [7, 69]) and TRPS1 (2–3%, [31, 37]), indicating that MGP is a good marker to differentiate breast cancer from lung adenocarcinoma.

We also found that MGP was positive in only 2 of 218 cases (0.9%) of urothelial carcinoma, which is known to be frequently labeled with GATA3 (70–90% [7, 70],). According to the documented literature and our data, the positivity of MGP, GATA3, and TRPS1 is extremely rare in other tumor types, such as cholangiocarcinoma and colorectal, gastric, and thyroid carcinomas. Ai et al. [31] found low TRPS1 expression in one melanoma, while none of the melanomas enrolled in our cohort was MGP-positive. Further investigation of MGP expression in other tumor types is needed, especially those for which relatively high TRPS1 or GATA3 expression has been reported, such as salivary duct carcinomas and pancreatic adenocarcinoma.

A limitation of this study is that the TMA samples we used may not be able to adequately represent the intra-tumor expression heterogeneity of the IHC markers [62, 63]. A multicenter prospective study using standard whole-tissue sections should be undertaken to fully validate the value of MGP in determining breast origin. Our study used a relatively higher number of total breast carcinomas and metaplastic breast carcinomas than the recently published studies to identify new breast cancer marker [31, 37, 38]. Nevertheless, more cases are included in our ongoing study to further evaluate the sensitivity and specificity of MGP in metastatic breast carcinomas, special type of invasive breast carcinomas, neuroendocrine neoplasms, salivary gland-type tumors (either primary in breast or salivary gland), as well as tumors exhibiting cartilaginous/osseous differentiation.

## Conclusion

Through bioinformatic analysis, we identified MGP as a novel IHC marker supporting breast origin, demonstrating relatively high sensitivity and specificity for invasive breast carcinoma of no special type. Further verification is needed for invasive breast carcinoma of special type as well as metaplastic breast carcinoma, especially those exhibiting cartilaginous/osseous differentiation. The joint application of MGP, TRPS1, and GATA3 could be recognized as a reliable diagnostic panel to determine breast origin in clinical practice.

## Supplementary Information

**Additional file 1: Fig. S1.** The mRNA levels of candidate genes in different molecular subtypes of breast cancer. ER/PR+ (ER/PR+ and HER−, *n* = 685), HER2+ (*n* = 168), and TNBC (*n* = 177). TCGA BRCA ER, PR, and HER2 status were retrieved from Thennavan et al. [21].

**Additional file 2. Table S1.** No. of cases in each TCGA tumor type.

**Additional file 3**. **Table S2.** Expression change of 17 genes in paired breast cancers and their metastases

**Additional file 4**. **Table S3.** Correlation between protein and mRNA levels of 9 candidate genes.

**Additional file 5**. **Table S4.** The immunoreactive scores of GATA3, TRPS1 and MGP in 1201 cases of breast cancer.

## Data Availability

The datasets supporting the conclusions of this article are available in the supplementary tables. Processed data and R codes were available from the corresponding author upon reasonable request.

## Abbreviations

MGP: Matrix Gla protein
GATA3: GATA-binding protein 3
TRPS1: Trichorhinophalangeal syndrome type 1
IHC: Immunohistochemistry
ER: Estrogen receptor
PR: Progesterone receptor
HER2: Human epidermal growth factor receptor-2
TNBC: Triple-negative breast cancer
TCGA: The Cancer Genome Atlas
CPTAC: Clinical Proteomic Tumor Analysis Consortium
TMA: Tissue microarray
FFPE: Formalin-fixed paraffin-embedded
PAM50: Prediction Analysis of Microarray 50
DE: Differential expression
FDR: False discovery rate
GI tract: Gastrointestinal tract
MBC: Metaplastic breast carcinoma
NST: No special type
SqCC: Squamous cell carcinoma
SpCC: Spindle cell carcinoma
FMC: Fibromatosis-like metaplastic carcinoma
MBC-MD: Metaplastic breast carcinoma with mesenchymal differentiation
