# Supplementary figures and images for: Matrix Gla protein (MGP), GATA3, and TRPS1: a novel diagnostic panel to determine breast origin
Source: Breast Cancer Res. 2022 Oct 25;24:70. doi: 10.1186/s13058-022-01569-1 (PMC9598034; doi:10.1186/s13058-022-01569-1)

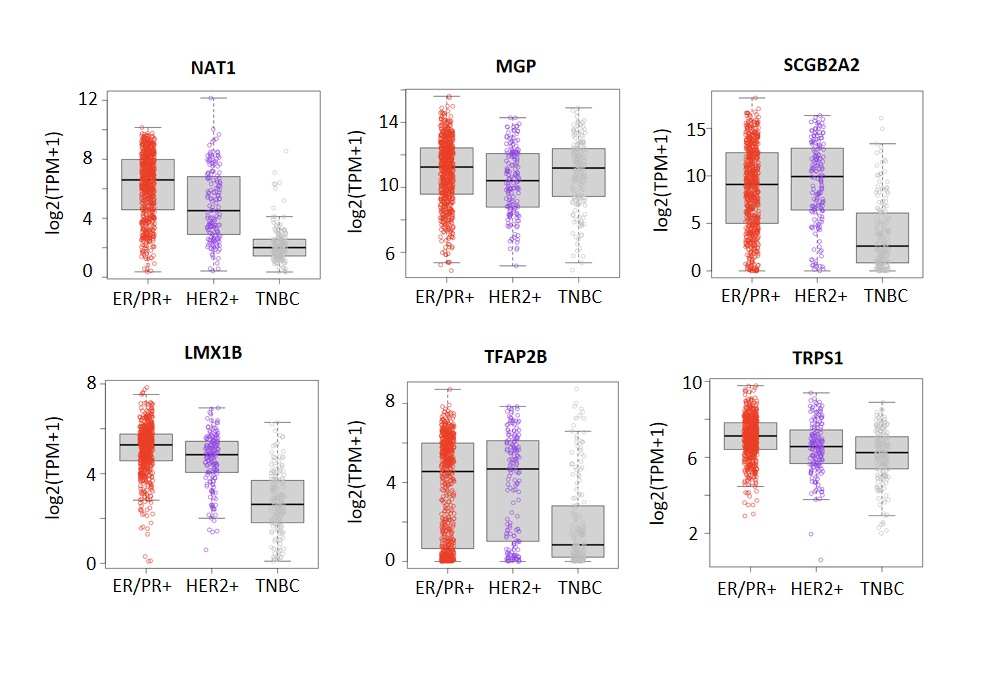

Supplement: Supplementary file 1 — Additional file 1: Fig. S1. The mRNA levels of candidate genes in different molecular subtypes of breast cancer. ER/PR+ (ER/PR+ and HER−, n = 685), HER2+ (n = 168), and TNBC (n = 177). TCGA BRCA ER, PR, and HER2 status were retrieved from Thennavan et al. [21]. [file 13058_2022_1569_MOESM1_ESM.jpg]
