# Supplementary material for: Matrix Gla protein (MGP), GATA3, and TRPS1: a novel diagnostic panel to determine breast origin
Source: Breast Cancer Res. 2022 Oct 25;24:70. doi: 10.1186/s13058-022-01569-1 (PMC9598034; doi:10.1186/s13058-022-01569-1)
Supplement: Supplementary file 2 — Additional file 2. Table S1. No. of cases in each TCGA tumor type. [file 13058_2022_1569_MOESM2_ESM.docx]

**Supplementary Table S1. No. of cases in each TCGA tumor type.**

| Tumor type | No. of cases | Up-regulated DE genes:  Breast vs each tumor type |
| --- | --- | --- |
| BRCA | 1119 | NA |
| ACC | 79 | 2514 |
| BLCA | 414 | 997 |
| CESC | 306 | 1483 |
| COAD | 483 | 1935 |
| DLBC | 48 | 3820 |
| GBM | 170 | 2109 |
| HNSC | 504 | 1673 |
| KICH | 66 | 3074 |
| KIRC | 542 | 1598 |
| KIRP | 291 | 1984 |
| LAML | 178 | 3325 |
| LGG | 532 | 2747 |
| LIHC | 374 | 2333 |
| LUAD | 541 | 661 |
| LUSC | 502 | 892 |
| OV | 430 | 1121 |
| PRAD | 502 | 1835 |
| READ | 167 | 2399 |
| SKCM | 472 | 1973 |
| STAD | 420 | 775 |
| THCA | 513 | 2506 |
| UCEC | 554 | 940 |
| UCS | 57 | 1207 |
| Overlapped |  | 33 |

Note: DE, differential expression; BRCA, breast invasive carcinoma; ACC, adrenocortical carcinoma; BLCA, bladder urothelial carcinoma; CESC, cervical squamous cell carcinoma and endocervical adenocarcinoma; COAD, colon adenocarcinoma; DLBC, lymphoid neoplasm diffuse large B-cell lymphoma; GBM, glioblastoma multiforme; HNSC, head and neck squamous cell carcinoma; KICH, kidney chromophobe; KIRC, kidney renal clear cell carcinoma; KIRP, kidney renal papillary cell carcinoma; LAML, acute myeloid leukemia; LGG, brain lower grade glioma; LIHC, liver hepatocellular carcinoma; LUSC, lung squamous cell carcinoma; OV, ovarian serous cystadenocarcinoma; PRAD, prostate adenocarcinoma; READ, rectum adenocarcinoma; SKCM, skin cutaneous melanoma; STAD, stomach adenocarcinoma; THCA, thyroid carcinoma; UCEC, uterine corpus endometrial carcinoma; UVM, uveal melanoma.
